# Standardized mean differences in meta‐analysis: A tutorial

**DOI:** 10.1002/cesm.12047

**Published:** 2024-03-10

**Authors:** Daniel Gallardo‐Gómez, Rachel Richardson, Kerry Dwan

**Affiliations:** ^1^ Department of Physical Education and Sport, University of Seville, Seville Spain. Epidemiology of Physical Activity and Fitness Across Lifespan (EPAFit) Research Group Seville Spain; ^2^ Cochrane Methods Support Unit, Evidence Production and Methods Department Cochrane London UK; ^3^ Department of Clinical Sciences, Liverpool School of Tropical Medicine Centre for Evidence Based Medicine Liverpool UK

## Abstract

This tutorial focuses on standardized mean differences (SMD) as effect measures in meta‐analyses. We will explain what they are, when they should be used, how to correctly compute and interpret them, and some of the most common error made within evidence synthesis.

## Graphical abstract

This tutorial focuses on standardized mean differences (SMD) as effect measures in meta‐analyses. We will explain what they are when they should be used, how to correctly compute and interpret them, and some of the most common errors made within evidence synthesis. Additionally, we have created a micro‐learning module to accompany this article which includes a real‐life example of meta‐analysis of SMDs. Moreover, there is the opportunity to test out how to use a tool to correctly calculate SMDs, and how to interpret them.

Standardised mean difference micro learning module

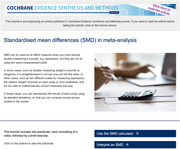

## 1 INTRODUCTION

In this tutorial, we focus on standardized mean differences (SMD); what they are, when they should be used, how to correctly compute and interpret them, and some of the common errors made within systematic reviews.

**Figure 1:**
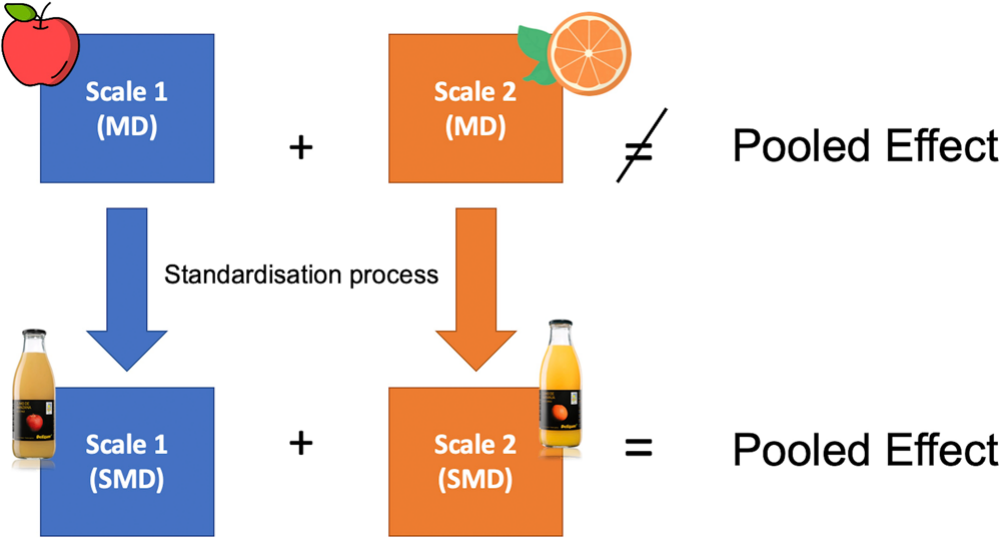
How standardized mean differences (SMDs) enable us to combine different scale‐specific units into one pooled effect. The illustration shows how data standardization process allows us to mix “apples” and “oranges” making them “juice.” MD, mean difference.

Review authors use the SMD as a summary statistic in meta‐analyses of continuous outcomes when the studies all assess the same outcome but measure it using a variety of different scales [1]. For example, we can look at a meta‐analysis in which included studies have measured the depressive symptoms of their participants using different scales/questionnaires [2] (e.g., the Beck Depression Inventory, the Geriatric Depression Scale, the Hamilton Rating Scale, or the Montgomery‐Asberg Depression Scale). From a statistical perspective, it is virtually impossible to quantitatively synthesize the results directly. Instead, a solution would be standardizing all these data to a common effect size measure: the SMD (Figure 1).

## 2 HOW TO COMPUTE STANDARDIZED MEAN DIFFERENCES

When we standardize data, we divide the mean difference (MD) between the treatment and control groups (i.e., the effect size of the treatment) by the pooled sample standard deviation (SD) in each study (i.e., the between‐participant variability in outcome measurements observed in each study) at one specific follow‐up time point [3]. Importantly, reviewers ought to fully detail these data management processes (e.g., data standardization) to ensure transparency and reproducibility in evidence syntheses [3].

(1) $$\mathrm{SMD}=\frac{\mathrm{MD}\mathrm{between}\mathrm{groups}}{\mathrm{SD}\mathrm{of}\mathrm{outcome}\mathrm{among}\mathrm{participants}\mathrm{at}\mathrm{follow}-\mathrm{up}\mathrm{time}\mathrm{point}}.$$

SMD calculation using pooled sample SD at a specific follow‐up time point.

For a better understanding of this terminology, we are going to apply different standardization methods on data extracted from a published meta‐analysis [4]. Therefore, we select the Ortiz‐Alonso et al. study [5] included in this meta‐analysis, which reported results using the overall score of the Short Physical Performance Battery (an instrument to assess the physical function; SPPB), and extract the “raw” data (i.e., data directly extracted from the study without any transformation) (Table 1).

**Table 1:** “Raw” data extracted from Ortiz‐Alonso et al. [5] study.

Study	Scale	Study arm	Baseline			Posttreatment		
			*N*	Mean	SD	*N*	Mean	SD
Ortiz‐Alonso et al. [5]	Short Physical Performance Battery	Treatment	143	4	2.5	125	3.2	2.5
		Control	125	4.2	3.1	125	3.8	2.9

Abbreviations: *N*, sample size; SD, standard deviation.

Next, using Equation (1), we standardize the data using the pooled SD of the outcome among participants at the posttreatment time point. First, we calculate both pooled sample SDs (baseline and posttreatment)

(2) $${\mathrm{SD}}_{\mathrm{pooled}}=\sqrt{\frac{{\mathrm{SD}}_{t}^{2}*\left(n_{t}-1\right)+{\mathrm{SD}}_{c}^{2}*\left(n_{c}-1\right)}{n_{t}+n_{c}-2}}.$$

Calculation of a pooled sample SD. The “*t*” suffix indicates treatment, and the “*c*” suffix refers to control arm.

Pooled sample SD at baseline time point:

$${\mathrm{SD}}_{\mathrm{pooled}}=\sqrt{\frac{2.5^{2}*\left(143-1\right)+3.1^{2}*\left(125-1\right)}{143+125-2}}=2.796.$$

Pooled sample SD at posttreatment time point:

$${\mathrm{SD}}_{\mathrm{pooled}}=\sqrt{\frac{2.5^{2}*\left(125-1\right)+2.9^{2}*\left(125-1\right)}{125+125-2}}=2.707.$$

Next, we convert arm‐based data into contrast‐based data (i.e., a single effect measure that summarizes the MD between the two study arms) using the below equations.

(3) $$\mathrm{MD}={\mathrm{Mean}\mathrm{score}}_{\mathrm{treatment}}-{\mathrm{Mean}\mathrm{score}}_{\mathrm{control}}$$

MD computation at posttreatment time point.

(4) $${\mathrm{SD}}_{\mathrm{MD}}=\sqrt{\frac{n_{t}+n_{c}}{n_{t}*n_{c}}*\frac{{\mathrm{SD}}_{t}^{2}*\left(n_{t}-1\right)+{\mathrm{SD}}_{c}^{2}*\left(n_{c}-1\right)}{n_{t}+n_{c}-2}}.$$

Computation of the SE of the MD at posttreatment time point.

$$\mathrm{MD}=3.2-3.8=-0.6,{\mathrm{SD}}_{\mathrm{MD}}=\sqrt{\frac{125+125}{125*125}*\frac{2.5^{2}*\left(125-1\right)+2.9^{2}*\left(125-1\right)}{125+125-2}}=0.342$$

Then, we standardize our MD and SE dividing them by the corresponding pooled sample SDs. Methodologists support the use of pooled SDs at baseline over follow‐up SDs, but it is common that studies only report follow‐up data. Therefore, we are going to standardize data in both cases: (1) supposing that we have baseline data, and (2) supposing that we only have follow‐up data.

Standardized data (SMD and SE) using the pooled sample SD at baseline time point.

$$\mathrm{SMD}=\frac{\mathrm{MD}}{{\mathrm{SD}}_{\mathrm{pooled}}}=\frac{-0.6}{2.796}=-0.215,{\mathrm{SE}}_{\mathrm{SMD}}=\frac{\mathrm{SE}}{{\mathrm{SD}}_{\mathrm{pooled}}}=\frac{0.342}{2.796}=0.122.$$

Standardized data (SMD and SE) using the pooled sample SD at posttreatment time point.

$$\mathrm{SMD}=\frac{\mathrm{MD}}{{\mathrm{SD}}_{\mathrm{pooled}}}=\frac{-0.6}{2.707}=-0.222,{\mathrm{SE}}_{\mathrm{SMD}}=\frac{\mathrm{SE}}{{\mathrm{SD}}_{\mathrm{pooled}}}=\frac{0.342}{2.707}=0.126.$$

Although this method is the most common applied in meta‐analyses, the use of a fixed scale‐specific SD reference is recommended [6, 7]. A more‐in‐depth explanation of this method can be found in Gallardo‐Gómez et al. [3] and the online content.

## 3 HOW TO INTERPRET STANDARDIZED MEAN DIFFERENCES

The SMDs express the size of the treatment effect in each study relative to the variability observed in that study. However, the overall treatment effect could be difficult to interpret as it is reported in units of standard deviation rather than the original units of measurement. Without guidance, clinicians and patients may have little idea how to interpret results presented as SMDs. There are two possibilities for **re‐expressing** such results in more helpful ways:

– Re‐expressing SMDs using **rules of thumb** for effect sizes. One example based on Cohen [8] is as follows: 0.2 represents a small effect; 0.5 a moderate effect: and 0.8 a large effect. Nevertheless, some methodologists believe that such interpretations are problematic because the importance of a finding is context‐dependent and not amenable to generic statements [7].
– Re‐expressing SMDs using a **familiar instrument**. The second (and recommended) option is to re‐express the SMD in the units of one or more of the specific measurement instruments. This method could be performed by multiplying the SMD by a typical among‐person SD for a particular scale (e.g., an external SD reference from a large cohort or cross‐sectional study that matches the target population, an internal SD reference, or a pooled sample SD), preferably, the same used for data standardization [3]. In this way, using the original scale‐specific units, the clinical relevance and impact of a pooled treatment effect can be interpreted more easily. In our example, when authors pooled all effect sizes, they obtained a pooled treatment effect of SMD = 0.40 (95% confidence interval: 0.02–0.77). We then re‐express this effect size into SPPB units multiplying by the external SD reference for the study population (external SD reference = 3.14), obtaining a scale‐specific pooled effect of MD = 0.97 (95% CI: 0.06–2.42). Considering a predefined minimally clinically important difference of 1 point in the SPPB, [9] we could support the use of an intervention (physical activity in this case [4]) in a specific population due to its *clinically* meaningful benefit in the outcome of interest.

## 4 COMMON PITFALLS USING STANDARDIZED MEAN DIFFERENCES

a) **Unnecessary data standardization**. Reviewers do not need to standardize their data when there are not different scales assessing the outcome of interest. The belief that the term “effect size” is a synonym of “SMD” can lead to authors reporting the treatment effect in SMD units when it is not needed. One example of this is when only one study is reported in a forest plot; an SMD is not needed, and this should be reported as MD.
b) **Use of SEs rather than SDs to calculate SMDs**. As we have seen in Equation (1), we use the posttreatment pooled sample SD to calculate SMDs. Nonetheless, primary studies could wrongly report the SE of an assessment as the SD or not specify whether they are reporting SD or SE. A red flag for this could be a quite low SD (i.e., <1), though it is highly dependent on the score range of the specific scale. This mistake could lead to “effect size inflation” because when you use SEs to calculate SMDs, you are dividing the MD by a lower value of the truly corresponding one, obtaining a higher value. Therefore, if you obtain SMDs greater than one, you should check whether the SD or SE has been used.
c) **Combination of change from baseline and posttreatment effect measures**. Although mixing change from baseline and posttreatment outcomes is not a problem when it comes to meta‐analysis of MDs [7], they should not in principle be combined using SMDs. This is because the SDs used in standardizing posttreatment values reflect between‐person variability at a single time point, where SDs used in change scores standardization reflect variation in between‐person changes over time, so will depend on both within‐person (dependent on the length of time between measurements) and between‐person variability [7].
d) **Effect size direction**. There are scales where an improvement in the outcome is reflected by a reduction in the score (e.g., in our illustrative example, the less time spent in walking a distance, the better functional capacity). In addition, to interpret the magnitude of an effect, we must consider the specific outcome (e.g., a more negative effect could be positive if the review is investigating depressive symptoms, meaning a reduction in these symptoms). To correct an effect that is not in line with the direction of our meta‐analysis, we should multiply the effect size value by –1, (no modifications are needed for the SD), ensuring that all effects are in the same direction.
e) **No interpretation of SMDs**. A huge number of meta‐analyses often leave their effect estimates as SMDs, which can make interpretation difficult. We have talked about different available options to re‐express SMDs to more‐interpretable estimates above.

**RECOMMENDATIONS**

- To promote reproducibility and transparency in evidence synthesis, provide details about the direction of the scales and effect size values, and the method used for data standardization and re‐expression.
- Ease the interpretation of your results re‐expressing the pooled effect from SMD into a more familiar scale‐specific unit.
- Inspect SMDs greater than 1 SD units to ensure the correct data is included in the analyses.

## 5 ADDITIONAL INFORMATION

When we mention “effect size” in this tutorial, and in Cochrane Reviews that synthesized standardized mean differences, we are implicitly referring to an effect size known in social sciences as **Hedges’** ***g***, which is similar to the effect size so‐called **Cohen's** ***d*** with a small adjustment for small sample bias. These effect sizes (Hedges’ *g* and Cohen's *d*) use a pooled SD in the denominator, which is an estimate of the SD based on outcome data from both intervention groups, assuming that the SDs in the two study groups are similar [7]. In contrast, another effect size called **Glass’ delta** (Δ) uses only the SD from the comparator group, on the basis that if the experimental treatment affects between‐person variation, then such an impact of the treatment should not influence the effect estimate.

All these effect measures referred to as SMDs can be calculated by hand or in any statistical package. Statistical packages in R software include *metafor* [10], *esc* [11], or *compute.es* [12]. A hands‐on useful resource is the *bookdown* of Harrer et al., [13] which serves an accessible introduction into how meta‐analyses are covered, including different SMD calculation, and pooling methods with examples.

## 6 FURTHER READING AND ONLINE CONTENT

More information on SMDs, can be found in Chapter 6.5 of The Cochrane Handbook for Systematic Reviews of Interventions [1].

**Figure 2:**
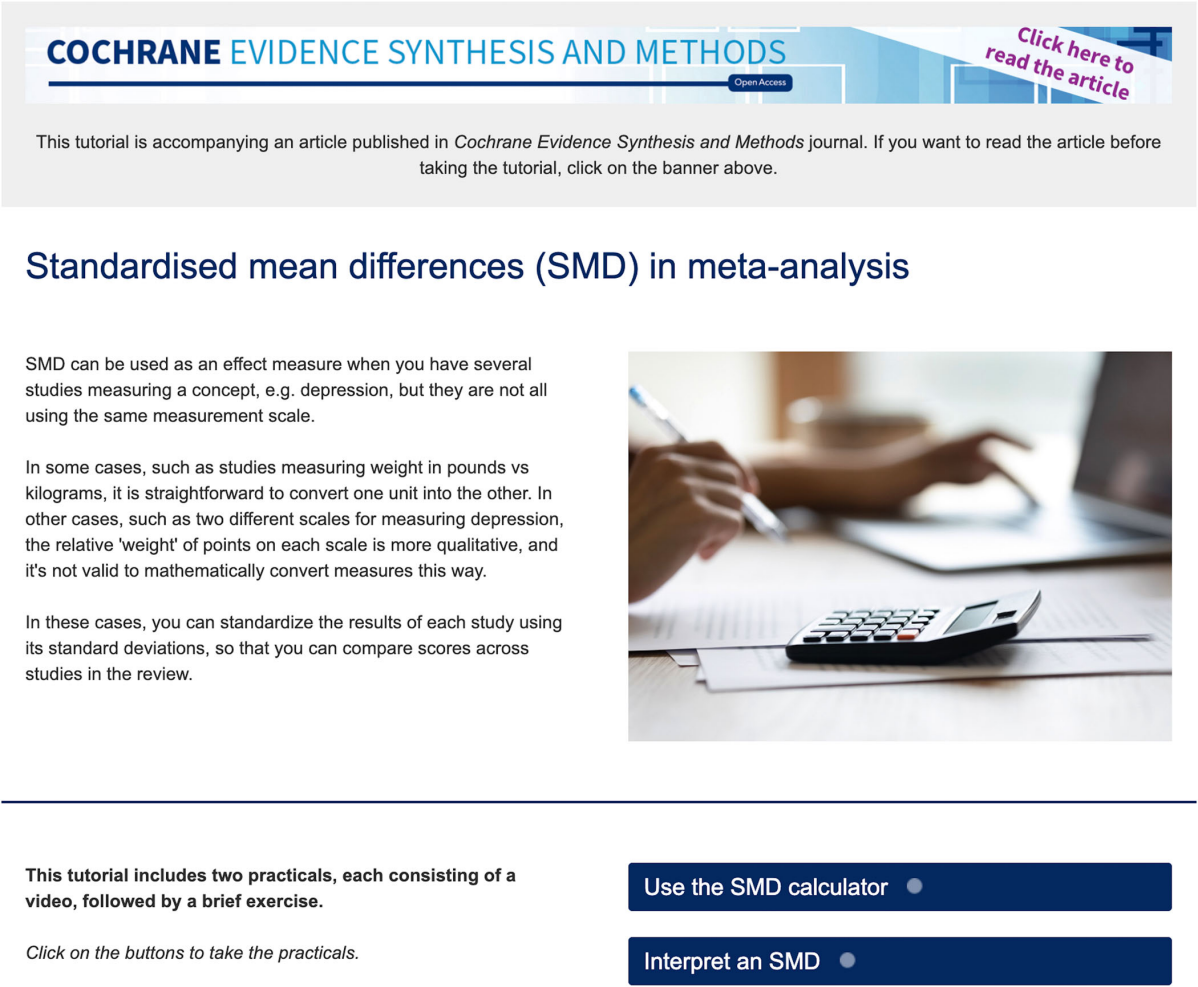
Screenshot of the micro‐learning module.

Cochrane Training have produced a micro‐learning module on how to calculate SMDs to accompany this article (https://share.gomolearning.com/sharelink/b17d5bf8ee76fd1056d6a2505eb81375793889d0773d0141fd/) (Figure 2).

## AUTHOR CONTRIBUTIONS

**Daniel Gallardo‐Gómez**: Conceptualization; writing—original draft; writing—review and editing. **Rachel Richardson**: Supervision; writing—review and editing. **Kerry Dwan**: Conceptualization; supervision; writing—review and editing.

## CONFLICT OF INTEREST STATEMENT

Rachel Richardson is employed by Cochrane. Kerry Dwan is a former employee of Cochrane. The other author declares no conflict of interest.

## PEER REVIEW

The peer review history for this article is available at https://www.webofscience.com/api/gateway/wos/peer-review/10.1002/cesm.12047.

## Data Availability

Data sharing is not applicable to this article as no data sets were generated or analyzed during the current study.
