# Correction to “Standardized mean differences in meta‐analysis: A tutorial”

**DOI:** 10.1002/cesm.12076

**Published:** 2024-06-02

In Equation 4 (computation of the standard error [SE] of the mean difference [MD] at posttreatment time point), we wrote SD instead of SE, which is the correct one (see attached figure below).


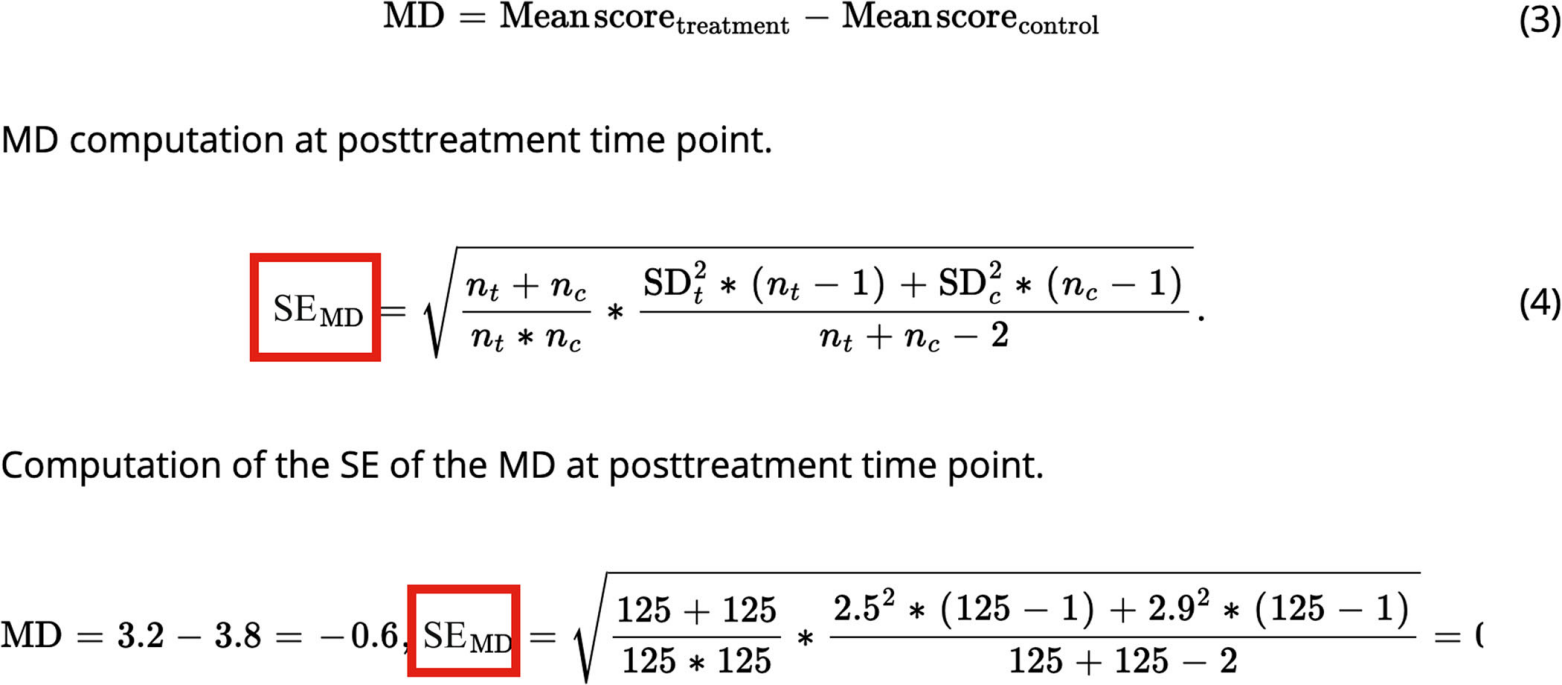


We apologize for this error.

